# Brain connectivity analysis based classification of obstructive sleep apnea using electroencephalogram signals

**DOI:** 10.1038/s41598-024-56384-9

**Published:** 2024-03-06

**Authors:** J. Rajeswari, M. Jagannath

**Affiliations:** 1 Department of Electronics and Communication Engineering, Agni College of Technology, Chennai, Tamil Nadu India; 2 School of Electronics Engineering, Vellore Institute of Technology (VIT) Chennai, Chennai, Tamil Nadu India

**Keywords:** ISRUC database, Brain connectivity, Wavelet packet decomposition, Delta band, Neuroscience, Health care, Medical research

## Abstract

Obstructive sleep apnea (OSA) is a disorder which blocks the upper airway during sleep. The severity of OSA will lead heart attack, stroke and end of life. This proposed study explored the classification of OSA and healthy subjects using brain connectivity analysis from electroencephalogram (EEG) signals. Institute of System and Robotics—University of Coimbra (ISRUC) database were used for acquiring 50 EEG signals using 4 channels and noise removal has been accomplished by 50 Hz notch filter. The Institute of System and Robotics—University of Coimbra (ISRUC) database contained 50 EEG signals, with four channels, and a 50 Hz notch filter was applied to remove noise. Wavelet packet decomposition method was performing the segregation of EEG signals into five bands; Gamma (γ), beta (β), alpha (α), theta (θ) and delta (δ). A total of 4 electrode positions were used for the brain connectivity analysis for each EEG band. Pearson correlation method was effectively used for measuring the correlation between healthy and OSA subjects. The nodes and edges were highlighted the connection between brain and subjects. The highest correlation was achieved in delta band of OSA subjects which starts from 0.7331 to 0.9172 respectively. For healthy subjects, the positive correlation achieved was 0.6995. The delta band has been correlated well with brain when compared other bands. It has been noted that the positive correlation well associated with brain in OSA subjects, which classifies OSA from healthy subjects.

## Introduction

The health of the brain can be controlled by enough quality sleep. However, sleep issues such as lack of sleep, obstructive sleep apnea (OSA), and insomnia are rather typical. The obstruction in the upper airway appears to be OSA, which is characterised by frequent cessations or reductions in airflow and can lead to intermittent hypoxia and fragmented sleep. In general, OSA leads to adverse systemic reactions such inflammation, hypertension, oxidative stress, and insulin resistance. It also has an impact on brain function and alters the architectural structure of the brain. The most common symptoms of the OSA is linked to reduced excessive daytime drowsiness through a number of processes, such as impaired blood–brain barriers, brain edema, and amyloid deposition, alterations in cerebral anatomy, altered anatomical brain connections, and blood—brain barrier failure^1^.

Through physical connections, distinct brain regions communicate with one another to create and combine information from various sources. The effectiveness of functional segregation and integration, as well as the connections and structural integrity of the brain, all have an impact on the structural network features^2^. The origination of OSA has been linked to aberrant central nervous system anatomy and function, particularly deficiencies in the insular cortex of the brain^3^. Less than 2% of the human insular cortex, which is connected to the limbic, temporal, cerebella and frontal regions have a variety of functions in cognition, speech production, and emotion processing, all of which are important OSA characteristics^4^. Activation of the insular cortex enhances control of the habenula on the raphe nucleus by reducing the genioglossus muscle tension, release of 5-hydroxytryptamine, and airway blockage. A meta-analysis highlighted the role of the insular cortex in neurocognitive, perceptual, somatosensory, and affective deficits in OSA individuals^5^. As a result, it's probable that impairments in the regional functional magnetic resonance imaging (fMRI) responses to emotions, evoked cognitive function, dyspnea, and sensorimotor function are the result of abnormalities in different insular sub regions. Studies on the functional activity of the brain in OSA patients have significantly highlighted deviations in the insula^1^. Fractional anisotropy (FA) measurement was suggested by Lee et al.^6^ as a way to check if people with OSA have changed brain network connections. Network irregularities may reflect brain tissue injury and be the mediating factor in clinical functional deficits.

In the discipline of neuroimaging, graph theoretical analysis has grown in popularity recently. It has been widely used to explain the topological properties of brain networks. Different types of network characteristics derived from the quantification technique of small-world architecture. For the human brain, the network quantification methods provide a unique structure that can be employed to scrutinize potential changes in the topological organization of the brain network^7^. It is feasible to decide the individualities of brain networks by picturing the human brain as a complicated network with nodes denoting areas of the brain that contain neurons and edges indicating neural connections^2^. According to earlier research, OSA patients' brains undergo structural changes as well as modifications to the connection of their functional modalities^8,9^, which may prompt us to look into potential modifications to brain structure correlation networks. The neuronal dynamics can be measured by the general neuroimaging instrument by clinicians from electroencephalogram (EEG) signals. The information processing of a person can be completed by the data taken from EEG results^10^. Latest technical advancements have expanded the range of EEG recording capabilities using the different arrays of electrodes connected to the skull, such as 128, 256, and 512. Frequency band separation is beneficial when particular sub-bands deliver the essential information. Enhanced frequency-band identification was accomplished while preserving good temporal resolution by using EEG pre-processing techniques with temporal resolution features and frequency band separation. EEG signals were separated into frequency bands using several techniques that delivered frequency and temporal resolution such as wavelet packet decomposition (WPD), wavelet transform (WT), discrete wavelet transform (DWT), and empirical mode decomposition (EMD) where the discovered frequency bands were employed as restrictions to resolve the inverse problem^11^. To investigate epilepsy detection, a model was developed, which included an attentional mechanism-based convolutional neural network model and an EEG-based multi-frequency multilayer brain network. To be more precise, the original EEG signals were divided into eight frequency bands using wavelet packet decomposition and reconstruction techniques, taking into account the multi-frequency properties of the brain. The correlation analysis between different brains regions was also used^12^.

### State of the art

Huang et al.^13^ used voxel-based morphology and the amplitude of low-frequency fluctuations analyses to compare cortical morphology and spontaneous brain activity obstructive sleep apnea (OSA) and healthy subjects, including 28 moderate-to-severe OSA and 34 healthy subjects, respectively. The Allen Human Brain Atlas and transcriptome-neuroimaging spatial correlation analysis were used for the investigation of gene expression patterns associated with changes in grey matter volume and amplitude of low-frequency fluctuations in OSA. Another study performed the mathematical analysis of functional connectivity in the EEG of patients with OSA and healthy subjects by calculating wavelet bicoherence from nighttime polysomnograms. After observing the interhemispheric synchronization deterioration, this study demonstrated that high-frequency EEG activity includes a compensatory increase in intrahemispheric connection and a modest increase in the connectivity of the central and occipital lobes^14^. OSA has been linked to structural brain changes in areas related to memory and Alzheimer's disease, and a recent study showed that OSA is becoming more widely acknowledged as a risk factor for cognitive decline. The study explained the substantial correlation between a higher apnea–hypopnea index and lower functional connectivity by the relationship between the medial prefrontal cortex and bilateral hippocampi, the left hippocampus, and the posterior cingulate cortex and precuneus^15^.

Long et al.^16^ described the significance of alterations in functional connectivity in the insular subregions and entire brain of patients receiving continuous positive airway pressure treatment. Additionally, the relationship between resting-state functional connectivity changes and cognitive impairment in OSA patients was explored. Research on OSA using limited channel EEG shows increased EEG slowing during resting wakefulness and deficiencies in slow wave activity and spindles during sleep. Additionally, local parietal impairments in delta power during non-rapid eye movement have been identified by high-density EEG^17^.

Based on the abovementioned methods, this study intended to explore the anatomical brain connection of OSA and healthy subjects chosen from an ISRUC database. Previous methods for network analysis have limitations in characterizing brain connections associated with OSA severity because it may be difficult to reconcile the relationship between a nonlinear system and uninterrupted metrics. The brain's functional connectivity between OSA and healthy subjects using EEG signals must show the severity. In this proposed study, the absolute values of wavelet co-efficient have been utilized to demonstrate the brain correlations between OSA and healthy subjects using the nodes and edges of the brain network. Initially, the procedure followed the frequency band separation of EEG signals, and a correlation matrix was implemented to discover the classification along with the nodes and edges of the brain.

## Methods and materials

### Database description

Institute of Sleep Research, University of Coimbra (ISRUC) dataset was used in this study which is publically available. Each recording was chosen at random from PSG recordings that have been collected from the special Medicine Center of the Hospital of Coimbra University for sleep^18^. Three categories were formed for dataset, 100 individuals, each with one recording session, 8 subjects, each with two recordings, and 10 healthy subjects, and each with one recording session. For this study, 25 subjects from group 1 and 25 subjects from groups 2 and 3 were selected to differentiate between obstructive sleep apnea (OSA) and healthy subjects. This included 40 OSA and ten healthy subjects. The Institute of System and Robotics—University of Coimbra (ISRUC) database contains electroencephalogram (EEG) signals from the F4-A1, F3-A2, O1-A2, and O2-A1 electrode positions were recorded and analyzed at a frequency of 200 Hz.

### Pre-processing and wavelet packet decomposition

After signal acquisition, a 50 Hz notch filter was used to eliminate the power line noise. In order to examine the brain activity during sleeping, the signals were subsequently filtered by a band pass filter operating between 0.5 and 35 Hz.

Followed by pre-processing, the procedure band separation was carried out using the wavelet packet decomposition (WPD). In comparison with wavelet decomposition, WPD decompose both low and high-frequency co-efficient of all the five bands of EEG such as alpha (α), theta (θ), gamma (γ), beta (β), and delta (δ) using (db4) wavelet with 5 level decomposition^19^. Assume that the wavelet basis function is ψ(t), and the scaling function is φ(*t*) (Eq. 1).

1 $$ W_{d} \left( t \right) = \mathop \sum \limits_{k} W_{j} \left( k \right)\varphi_{jk} \left( t \right) + \mathop \sum \limits_{k} D_{j} \left( k \right)\psi_{jk} \left( t \right) $$

*W*_*d*_(*t*) is the decomposed EEG signal, *W*_*j*_(*k*) is approximation coefficients, *D*_*j*_(*k*) is detail coefficients, *φ*_*jk*_(*t*) is scaling function, and *ψ*_*jk*_(*t*) is mother wavelets.

Brain connectivity analysis was utilized to categorize OSA and healthy participants by calculating the absolute value of the decomposed five band EEG signals, which were used as a feature. The brain connectivity analysis appears to be a classifier in this proposed study, and the absolute values of the five band EEG signals function as features.

### Brain connectivity analysis

Since the network is made up of nodes (brain regions) and edges (connection between nodes), the MATLAB toolbox BrainNet Viewer has been utilized for tracking the changes. Four electrode positions from the ISRUC database was utilized and 16 connections were made with positive correlation. The Broadmann Atlas has been used to verify each electrode's coordinates^20^. The total number of edges connecting each node is referred to as the node degree. Initially, the first adjacency matrices for each participant were used to determine the node degree. Therefore, significant node degrees were found in the two groups using null t-test results for the brain areas. To ascertain how well the significant hubs reflect the functional networks depicted in OSA and healthy groups, the null t-test findings were correlated with the network maps using Pearson correlation method. From the separated EEG bands, alpha, delta, theta and delta were used in this investigation to examine the brain connections. For each participant, the t values were computed in those four bands for connectivity analysis.

### Statistical analysis

The EEG signals in this study were found to be non-normally distributed. Therefore, non-parametric analysis was conducted using the Statistical Package for the Social Sciences (SPSS) for Windows, version 15.0. The Mann–Whitney-Wilcoxon test was used to identify significant differences between OSA and healthy subjects, with a significance level set at *p* = 0.05. The differences between delta, beta, theta, and alpha bands for OSA and healthy subjects were analyzed using the same test. The absolute value wavelet coefficients of delta and beta bands were reported to be significant (*p* < 0.05) in the middle frontal gyrus and the frontal and occipital poles.

## Results

**Figure 1:**
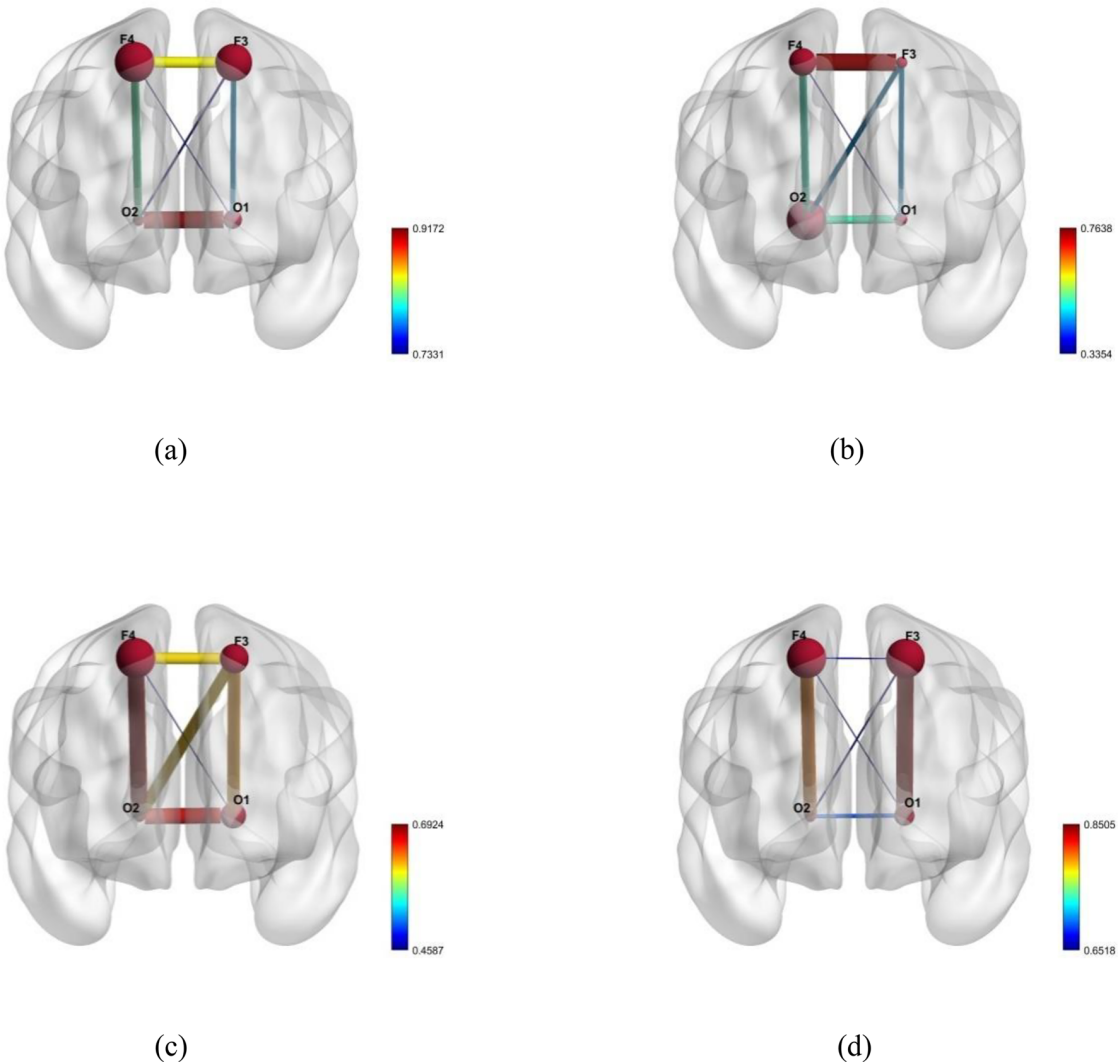
Topographical correlations in brain regions of obstructive sleep apnea subjects (**a**) Delta, (**b**) Theta, (**c**) Alpha and (**d**) Beta bands.

The obstructive sleep apnea (OSA) and healthy subjects from the ISRUC dataset were obtained for the purpose of conducting a brain connectivity analysis. Wavelet packet coefficient was used to generate the correlation matrix. Graph theoretical studies were used to track the nodes and edges of the brain connectivity of both the subjects. Figure 1a–d shows the coronal view of the scalp data for the delta, theta, alpha, and beta bands of the EEG signals collected from subjects with OSA.

The nodal strength seems to be high in left hemisphere of the brain for OSA subjects. While the nodes occipital (O2) and F4 were strong in theta and alpha bands, the left frontal nodes F4 and F3 exhibited enhanced node strength in both bands. The delta and beta bands demonstrated the minimum and maximum positive correlation values such as 0.7331 & 0.9172 and 0.6518 & 0.8505 respectively. Meanwhile the minimum and maximum positive correlation values for theta band are 0.3354 & 0.7654 respectively and 0.4587 & 0.6924 for alpha band. The activation regions were discovered from the anatomy of brain Atlas Label in FSL and the Harvard–Oxford Cortical Structural Atlas^21^ depicted in Table 1. In pre-treatment OSA, there has been evidence of increased hippocampus and left fronto-lateral activity as well as decreased right fronto-lateral activity^22^.

**Table 1:** Positions of the significant electrodes from Harvard–Oxford Cortical Structural Atlas and Atlas Label in FSL were used to label the positions of the electrodes on the scalp.

S. no.	Electrode position	Brain regions covered (%)	Broadmann area
1	F3	Frontal pole –(36), Middle frontal gyrus –(64)	Left—BA9
2	F4	Middle frontal gyrus –(47), Frontal pole –(53)	Right—BA9
3	O2	Occipital pole –(100)	Right—BA18

**Figure 2:**
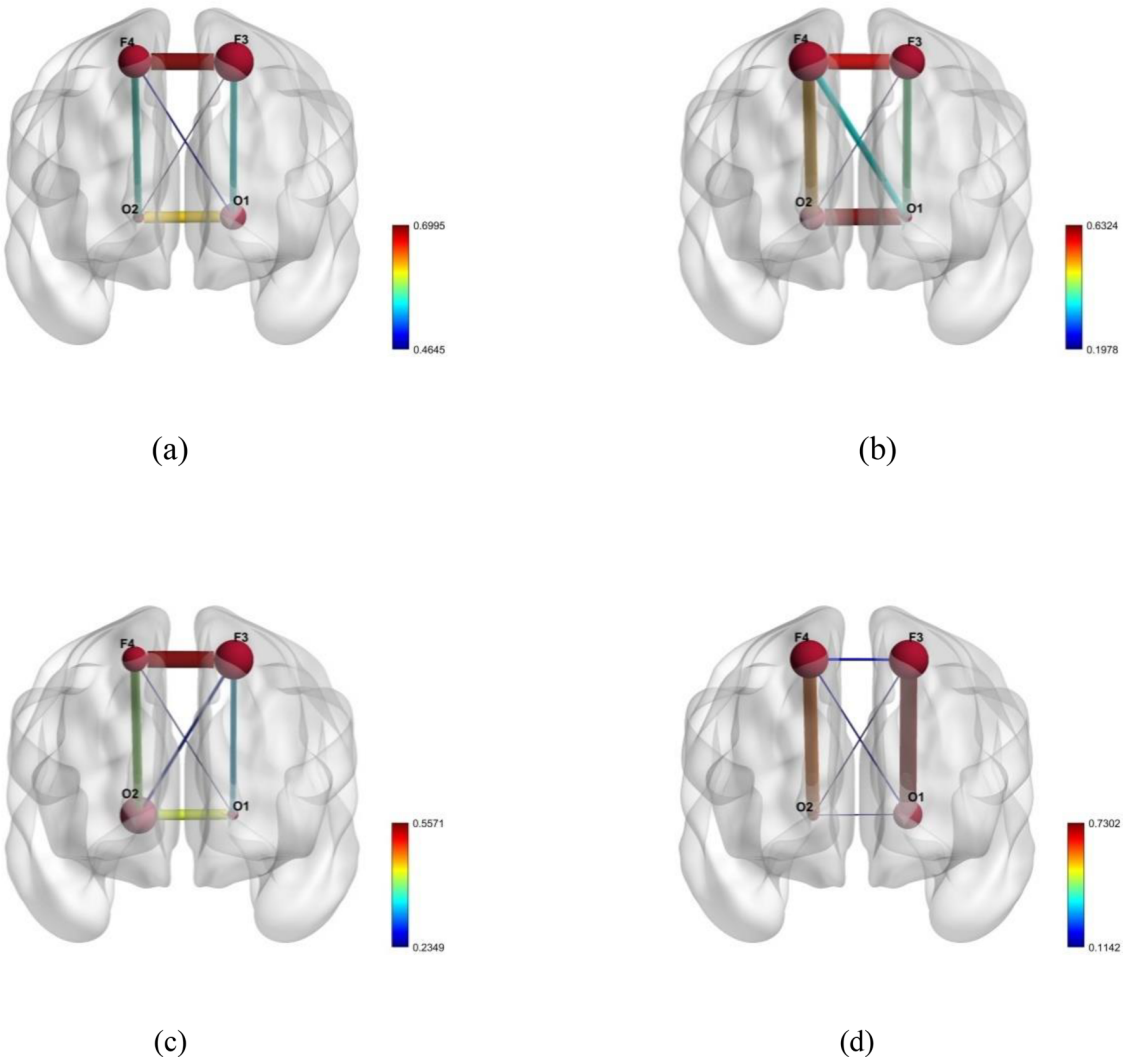
Topographical correlations in brain regions of healthy subjects (**a**) Delta, (**b**) Theta, (**c**) Alpha and (**d**) Beta bands.

Meanwhile, the minimum and maximum positive correlations appear to be relatively low (0.4645 and 0.6995, respectively, and 0.1142 and 0.7302) in the delta and beta bands of healthy subjects. Additionally, the minimum and highest positive correlation values for the alpha and theta bands were quite low; (0.1979 and 0.6324), (0.2349 and 0.5571) respectively that was depicted in Fig. 2. For healthy participants, the link was strongly present in the right hemisphere of the brain. Comparing OSA patients to healthy subjects, it was discovered that the middle frontal gyrus, the frontal pole, and occipital pole were correlated positively with brain.

**Figure 3:**
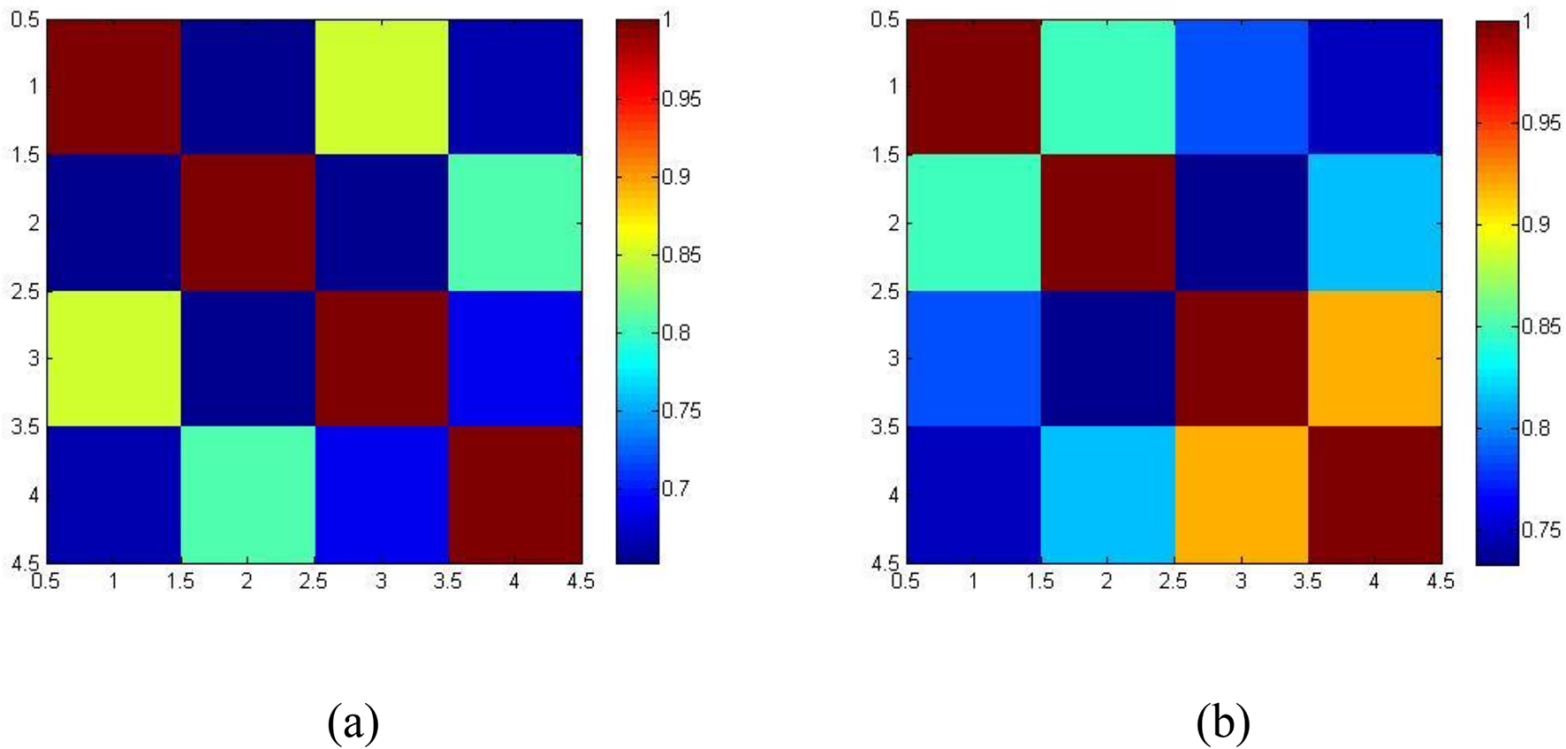
Correlation matrix for four brain lobes of obstructive sleep apnea subjects (**a**) Beta, (**b**) Delta bands.

The correlation matrix of beta and delta band for OSA subjects was presented in Fig. 3a and b, which shows the correlation with lobes and their connectivity with brain. In Fig. 3b, the x-axis range from 2.5 to 4.5 seems to have a higher correlation in values 0.9172 for the delta band, indicating occipital regions (O1 and O2). Consequently, in Fig. 3a the x and y axes range from 2.5 to 3.5. The frontal (F3) and occipital (O1) lobe moderately correlated with this region, which denotes 0.8505 for the beta band.

**Figure 4:**
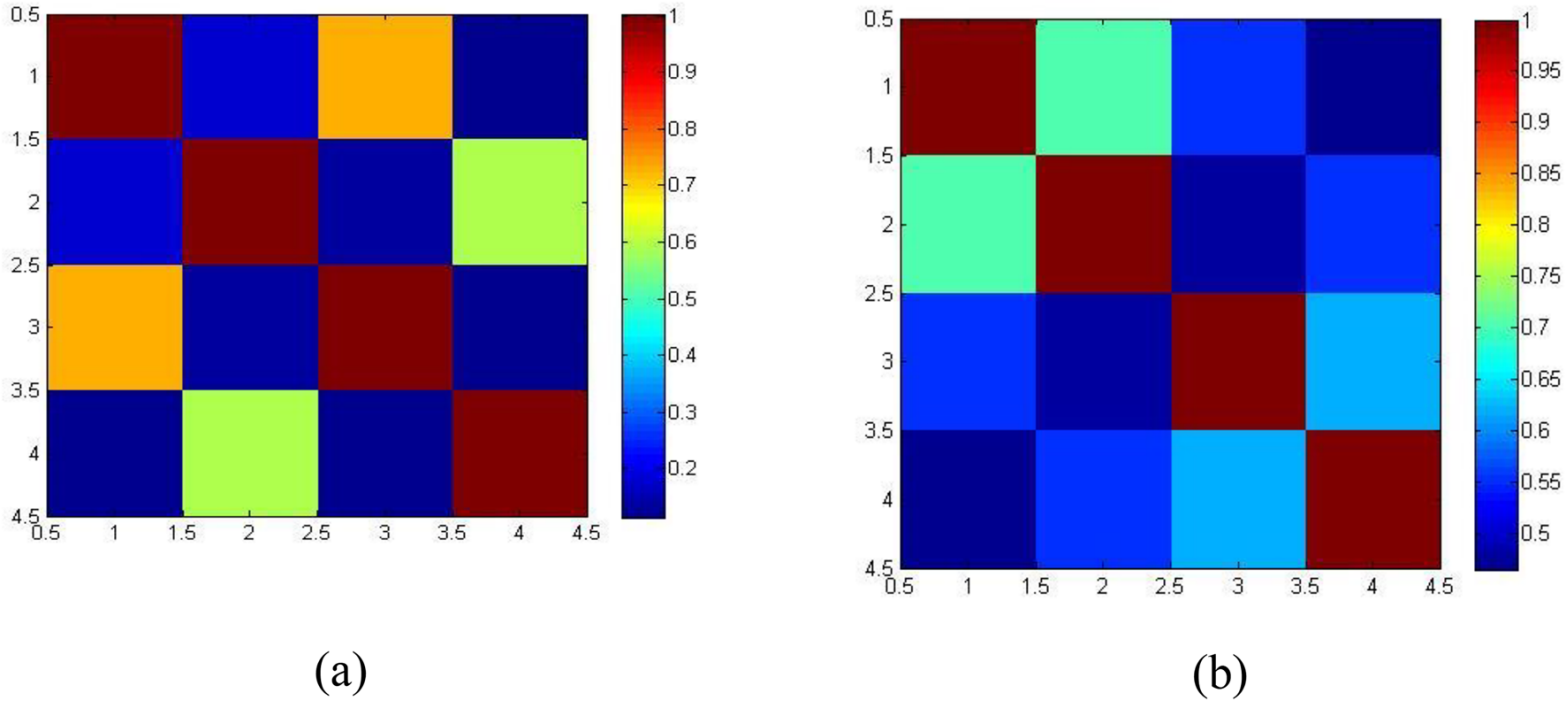
Correlation matrix for four brain lobes of healthy subjects (**a**) Beta, (**b**) Delta bands.

To compare the correlation matrix with OSA, the healthy subject’s connectivity also depicted in Fig. 4a and b. This will lead to the conclusion that the delta and beta bands were indeed connected with the brain on OSA subjects because the correlation values in healthy subjects appear to be rather low comparatively.

Figure 4a and b present the correlation matrix for healthy subjects. In Fig. 4b, there is a strong correlation between the x and y axes, ranging from 1.5 to 2.5, for the delta band, with a value of 0.6995. In Fig. 4a, for the beta band, the values range from 1.5 to 2.5 and show a correlation of 0.7302.

When comparing individuals with obstructive sleep apnea (OSA) to those who are healthy, it was found that there is a significant correlation between the values in the delta band. The study showed that there is a strong correlation between the brain connections of people with OSA and healthy individuals in the delta band, specifically in the frontal and occipital regions.

## Discussion

In this study, the functional connection of the brain between people with OSA and healthy subjects was presented. The wavelet packet decomposition was used to segregate the EEG signals bands and absolute values of the wavelet packet co-efficient were utilized for correlation study (Pearson correlation). Many neuropsychiatric illnesses may be brought on by malfunction in the functional networks that make up the brain, which are thought to be generated by dynamically connected areas that interact with one another to carry out specific activities^23^. The existing study recognized that, OSA patients exhibited higher left fronto-lateral action and decreased right fronto-lateral action through working memory tasks compared to normal subjects^22^. Luo et al.^7^ indicated a tendency for change that was more pronounced in the right hemisphere of OSA, which may be supported by a number of earlier studies from different modalities, including as resting-state functional MRI (rs-fMRI), and positron emission tomography. In this proposed study, the right hemisphere of study participants with OSA showed a strong positive connection. In slow-wave sleep, Horovitz et al.^24^ observed an interruption of association in the prefrontal cortex, while the Larson-Prior recognized a rise in the functional connectivity in the dorsal attention network during light sleep^25^. While slow waves have been shown to originate in the cortex and play a crucial role in their synchronization, sleep spindles are produced in the thalamus through interactions between neurons in the thalamic reticular nucleus and neurons in the thalamo cortex^26,27^. These observations highlight the delta band activation in those with OSA when combined with the existing studies as shown in Fig. 1, which evident the slow wave sleep occurred in OSA subjects.

In addition, the frontal lobe is a critical part of the brain that performs a variety of cognitive tasks. It may help to understand the resting state functional connectivity between the left vertebral artery and left inferior frontal gyrus in this study demonstrated a substantial negative correlation with visuospatial/executive scores by considering that abnormal resting state functional connectivity including as deficits in working memory, attention, learning, and executive skills, may be related to the amygdala-frontal lobe connection^28^. Another study conducted the correlation analyses revealed a positive correlation between Rey's auditory verbal learning test scores and sleep events (Rapid eye movement and N3), which may be a significant contributing factor to the reduction in episodic memory and learning capacities. Huang et al.^29^ identified a modification between the frequency band estimation and the classification accuracy of the EEG functional connectivity configuration in the beta1 frequency band. In the evaluation of frequency bands, the beta1 band has fewer chosen features than the alpha band and has very high discriminative ratios between rapid eye movement (REM) and N2 and REM and N3, although classification accuracy is typically poor.

Brain connectivity analysis has mainly been used to monitor memory tasks, showing stronger activation in the left frontal region and lower activation in the right frontal region. According to several research studies, persons with OSA appear to have high activity in their right hemisphere. In contrast, the prefrontal cortex was actively larger during light sleep. Also, patients with OSA exhibited the most increased delta band activation, according to certain studies. While in the rapid eye movement (REM) state, the beta1 band also improved performance. According to the abovementioned results, this proposed study obtained brain activation in frontal lobes F3 and F4 in both OSA and healthy subjects, which is positively correlated. Compared to healthy subjects, the OSA subjects showed higher brain activation in delta and beta bands than in alpha and theta bands. The brain connectivity analysis used in this proposed study produced improved results in the right hemisphere region of the brain. Specifically, the frontal and occipital lobes of OSA subjects were actively connected, and there appeared to be a higher correlation between frequency bands in the beta and delta bands than in the healthy subjects. As a result, the brain connectivity analysis is a prominent classifier for OSA subjects.

## Conclusions

This study used brain connectivity analysis to classify subjects with Obstructive Sleep Apnea (OSA) and healthy subjects. The analysis was based on brain networks using the Pearson correlation function. Additionally, wavelet packet decomposition was used to determine which band correlated strongly with the brain and its lobes. The results showed that the delta and beta bands of OSA activation had a higher correlation with the brain than healthy subjects. Although the results of the present study exhibit promise, the generalizability and robustness of the approach could be further validated with a more extensive and diverse dataset. Expanding the dataset and incorporating real-time monitoring allows us to refine and optimize the proposed approach for more robust and clinically applicable OSA classification.

## Data Availability

Data available in a publicly accessible repository. The data presented in this study are openly available in [https://sleeptight.isr.uc.pt/?page_id=48].
